# Acute and subchronic exposure to air particulate matter induces expression of angiotensin and bradykinin-related genes in the lungs and heart: Angiotensin-II type-I receptor as a molecular target of particulate matter exposure

**DOI:** 10.1186/s12989-015-0094-4

**Published:** 2015-06-26

**Authors:** Octavio Gamaliel Aztatzi-Aguilar, Marisela Uribe-Ramírez, José Antonio Arias-Montaño, Olivier Barbier, Andrea De Vizcaya-Ruiz

**Affiliations:** Departamento de Toxicología, Centro de Investigación y de Estudios Avanzados del Instituto Politécnico Nacional, Avenida Instituto Politécnico Nacional, 2508, México D. F, CP. 07360 Mexico; Departamento de Fisiología, Neurociencias y Biofísica, Centro de Investigación y de Estudios Avanzados del Instituto Politécnico Nacional, Avenida Instituto Politécnico Nacional, 2508, México D. F, C.P. 07360 Mexico

**Keywords:** Renin-angiotensin-aldosterone system, Kallikrein-kinin system, Particulate matter

## Abstract

**Background:**

Particulate matter (PM) adverse effects on health include lung and heart damage. The renin-angiotensin-aldosterone (RAAS) and kallikrein-kinin (KKS) endocrine systems are involved in the pathophysiology of cardiovascular diseases and have been found to impact lung diseases. The aim of the present study was to evaluate whether PM exposure regulates elements of RAAS and KKS.

**Methods:**

Sprague–Dawley rats were acutely (3 days) and subchronically (8 weeks) exposed to coarse (CP), fine (FP) or ultrafine (UFP) particulates using a particulate concentrator, and a control group exposed to filtered air (FA). We evaluated the mRNA of the RAAS components *At1*, *At2r* and *Ace,* and of the KKS components *B1r*, *B2r* and *Klk-1* by RT-PCR in the lungs and heart. The ACE and AT_1_R protein were evaluated by Western blot, as were HO-1 and γGCSc as indicators of the antioxidant response and IL-6 levels as an inflammation marker.

We performed a binding assay to determinate AT_1_R density in the lung, also the subcellular AT_1_R distribution in the lungs was evaluated. Finally, we performed a histological analysis of intramyocardial coronary arteries and the expression of markers of heart gene reprogramming (*Acta1* and *Col3a1*).

**Results:**

The PM fractions induced the expression of RAAS and KKS elements in the lungs and heart in a time-dependent manner. CP exposure induced *Ace* mRNA expression and regulated its protein in the lungs. Acute and subchronic exposure to FP and UFP induced the expression of *At1r* in the lungs and heart. All PM fractions increased the AT_1_R protein in a size-dependent manner in the lungs and heart after subchronic exposure. The AT_1_R lung protein showed a time-dependent change in subcellular distribution. In addition, the presence of AT_1_R in the heart was accompanied by a decrease in HO-1, which was concomitant with the induction of *Acta1* and *Col3a1* and the increment of IL-6. Moreover, exposure to all PM fractions increased coronary artery wall thickness.

**Conclusion:**

We demonstrate that exposure to PM induces the expression of RAAS and KKS elements, including AT_1_R, which was the main target in the lungs and the heart.

## Background

Air quality has been associated with increases in the morbidity and mortality due not only to pulmonary diseases but also to cardiovascular diseases. While the first group of diseases can be explained by the fact that the respiratory system is the major route of exposure to air pollutants, the second group could be a consequence of intermediate steps that connect lung damage to cardiovascular alterations . Epidemiological studies indicate that particulate matter (PM) is involved in these diseases and that exposure to particulate less than 2.5 μm is a major risk factor for the promotion and exacerbation of pulmonary and heart diseases [1–4].

The main mechanisms proposed to explain PM-induced toxic effects on human health are inflammation and oxidative stress. Recently, it has been demonstrated that the inflammatory response to PM promotes accelerated clotting with the participation of IL-6 cytokine [5]. Furthermore, oxidative stress can decrease heart rate variability after acute PM exposure [6]. Both mechanisms are involved in the pathophysiology of lung and heart diseases, i.e., COPD, asthma, fibrosis, hypertension, atherosclerosis, thrombosis, heart failure, etc., and are able to regulate or promote physiological responses including the activation of macrophages and neutrophils, regulation of the vascular tone, and production of mediators of signaling pathways, amongst others.

Important endocrine systems that regulate cardiovascular physiology are the Renin-Angiotensin-Aldosterone system (RAAS) and the Kallikrein-Kinin system (KKS).

Both systems are composed of peptidase enzymes [angiotensin-I converting enzyme type-1 and -2 (ACE and ACE2, respectively) and tissue kallikrein (KLK-1)]; and receptors [angiotensin receptors type-1 and type-2 (AT_1_R and AT_2_R, respectively); and bradykinin receptors type-1 and type-2 (B_1_R and B_2_R)]; and peptide precursors [angiotensinogen and kininogen (ANG and KNG, respectively)] [7–10]. Both systems control cardiac muscle contraction through vasoactive peptides, RAAS induces vasoconstriction through the ACE/Ang-II/AT_1_R axis, but by itself, RAAS can induce vasodilatation by the stimulation of NO-bradykinin dependent release through ACE2/Ang-(1-7)/Mas receptor axis [11], which also, in cooperation with B_2_R, AT_2_R induces a vasodilatation effect. This effect orchestrated by B_2_R, increases the release of NO through the activation of endothelial oxide nitric synthase (eNOS) [8, 12]. Vascular dilatation has been reported to be dependent of KLK-1, B_2_R and AT_2_R [13], where KLK-1 has an important role in the generation of the vasodilator peptides (bradykinin and kallidin) [14, 15].

Moreover, angiotensin-II through the AT_1_R promotes the activation of NADH/NADPH oxidase and the production of superoxide anion radicals as second messengers [16, 17] as well as B_1_R receptor does when it recognizes the des-Arg-bradykinin [18, 19]. In this sense, AT_1_R activation induces the expression of B_1_R [20, 21] and both induce a vasoconstriction effect with an increment in blood pressure [19, 22].

Furthermore, during the inflammation process RAAS and KKS are active participants of this process, AT_1_R is up-regulated by TNFα [23], IL-6 [24] and IL-1α, but is down-regulated by TNFα and INFγ combined [25]. Ang-II, through AT_1_R, induces IL-6 release and is inhibited by pyrrolidine dithiocarbamate radical scavenger [26]. On the other hand there is not enough information about cytokines regulating AT_2_R expression. KKS induces allodynia through B_1_R as part of the role in hyperalgesia [18]; macrophages release TNFα and IL-1 through the B_1_R pathway [27]. In lung fibroblasts B_2_R activation induces the release of IL-6 and IL-8 [28], and also IL-1β through a NF-κB-dependent pathway [28]. In summary, both systems control cardiac muscle contraction, levels of nitric oxide [8, 29], and are involved in the inflammatory response [30, 31] and in the blood coagulation state [32, 33].

There is a sustained cross-talk and a counterbalance between RAAS and KKS through the activation and degradation of vasoactive peptides through angiotensin-converting enzyme-I (ACE), which activates angiotensin-I to angiotensin-II and degrades bradykinin to bradykinin _(1-5)_ [34–36]. It is important to note that one of the main metabolic functions of the lungs is the activation and degradation of these peptides [36, 37].

During pathological cardiovascular states, the RAAS is overexpressed, as demonstrated in diseases such as hypertension, atherosclerosis, heart infarction and heart failure. However, recent evidence also indicates its participation in the pulmonary fibrosis induced by bleomycin [38] and bronchial hyperresponsiveness in asthmatic patients [39].

PM exposure has been established as a risk factor for lung and cardiovascular diseases, and RAAS and KKS are involved in the pathophysiology of these afflictions. However, there is not currently enough evidence to confirm whether RAAS and KKS are involved in the response to PM exposure. Ulrich et al. demonstrated that the activity of angiotensin-converting enzyme (ACE) in plasma and its mRNA in the lung decreased after 4 or 7 days of exposure to ECH-93 PM and ozone [40]. Gunisson and Chen reported a 1.5-fold increase in the differential expression *of At1r* in a lung microarray of double-knockout mice (apoE ^-/-^ and LDLr^-/-^) exposed subchronically to ultrafine particulates (6 h/day; 5 days/week for 4 months) [41]. Li et al. observed *ex-vivo* vasoconstriction of pulmonary artery rings exposed to PM (1-100 μg/ml), the soluble fraction of the PM, and metals including copper and vanadium. This observation was associated with an activation of MAPK signaling. These effects were completely inhibited by losartan and partially with captopril, a blocker of AT_1_R and an inhibitor of ACE, respectively [42]. Other studies using a mouse model of long-term exposure to PM_2.5_ reported cardiac remodeling observed to be associated with a switch to fetal reprogramming, the induction of fibrosis and hypertrophic markers and the impairment of the myocardial contraction [43, 44]. These processes have been reported to be mediated by RAAS.

There is not currently sufficient evidence to establish whether PM exposure could affect expression of RAAS and KKS elements. We therefore hypothesized that PM regulates expression of RAAS and KKS elements in the lungs, and concurrently in the heart, and these changes are accompanied by antioxidant/inflammation and myocardial gene responses.

The aim of this work was to screen for changes in some RAAS- and KKS-related elements during *in vivo* acute and subchronic exposures to three main airborne particle fractions (coarse, fine and ultrafine particulates) and to examine: 1) whether the effect of PM on RAAS and KKS elements expression is present in the lungs and concomitantly in the heart, 2) if there is a relationship between the exposures and antioxidant responses (heme oxygenase-1 and gamma-glutamyl-cysteine ligase), and 3) tissue damage by histological evaluation and changes in fetal gene reprogramming.

## Results and discussions

### mRNA levels of angiotensin and bradykinin system related-genes in the lungs

To evaluate whether PM exposure affects the angiotensin and bradykinin systems as an initial response to the toxic effect of the three PM fractions, we evaluated the mRNA levels of three genes of each endocrine system.

We observed an increase in the *Klk-1* mRNA in the lungs of the groups acutely exposed to FP and UFP, which was the only response of the bradykinin system observed after either exposure. However, the *B1r* and *B2r* mRNA levels showed a tendency to increase after the acute exposure; in the subchronic exposure, we did not observe any response of the bradykinin system genes (Table 1).

**Table 1:** Particulate matter effects on gene expression of angiotensin and bradykinin systems in the lungs. Semi-quantitative expression results of angiotensin-receptor type-2 (*At2r*), bradykinin receptor type-1 (*B1r*) and type-2 (*B2r*), and kallikrein (*Klk-1*) enzyme after acute and subchronic exposures

Acute exposure				
Group	FA	CP	FP	UFP
Gene				
*At2r*	1	1.3	1.85	1.32
	(0.6-1.4)	(0.61-2.17)	(1.1-2.5)	(0.5-1.9)
*B1r*	1	1.05	1.3	1.08
	(0.9-1.1)	(0.8-1.2)	(1.01-2)	(0.9-1.1)
*B2r*	1.1	1.1	2	1.4
	(0.6-1.2)	(0.9-1.77)	(0.9-3.1)	(1.4-1.7)
*Klk-1*	1	1.12	1.88 *	1.43 *
	(0.94-1.1)	(0.76-1.3)	(1.4-2)	(1.1-2.35)
Subchronic exposure				
Group	FA	CP	FP	UFP
Gene				
*At2r*	1	0.73	0.85	0.82
	(0.8-1.15)	(0.7-1.1)	(0.5-1.1)	(0.6-1.1)
*B1r*	1	0.95	1.	1
	(0.9-1.2)	(0.9- 1.03)	(0.94-1.5)	(0.93-1.1)
*B2r*	1	1.03	1.6	1
	(0.9-1.2)	(1-1.2)	(1.15-4.2)	(0.93-1.1)
*Klk-1*	1	0.84	1.78	0.92
	(0.8-1.2)	(0.8-1.1)	(0.7-2.7)	(0.9-1.4)

Acute exposure was defined as 5 h per day for 3 days

Subchronic exposure was defined as 5 h per day, 4 days per week, for 8 weeks

All results were corrected using glyceraldehyde 3-phosphate dehydrogenase as a housekeeping gene

AF: Air filtered

CP: Coarse particulate

FP: Fine particulate

UFP: Ultrafine particulate

Data are showed as median and range

* Indicates significantly different from FA

Our results demonstrate that, in the lungs, the KKS system responds only after an acute exposure, and the main gene that responded to airborne particulate was *Klk-1.* This is a serine protease enzyme that is necessary to produce kinin peptides. On this basis, particulate less than 2.5 μm could interfere with the activation of kinin peptides, which are necessary to activate bradykinin receptors. Bradykinin receptors are involved in the regulation of nitric oxide, the main vasodilator molecule, and are implicated in inflammation and vascular tone regulation. The expression of the bradykinin receptors in the lungs was not significantly altered for any of the exposures (regardless of particle type or duration of exposure). This could have been because both receptors are constitutively expressed in the lung tissue, although they can be induced by some cytokines including IL-1 and TNFα. PM exposure is characterized by the induction of cytokine release [8], but we did not observe changes in the expression of these receptors.

**Fig. 1:**
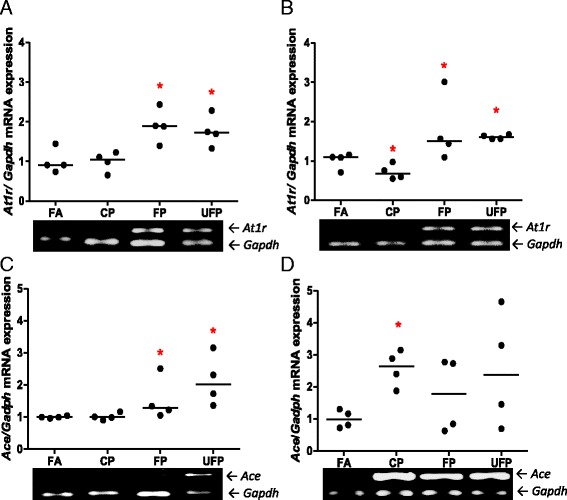
Particulate matter induces lung AT_1_R and ACE mRNA in a size- and time-dependent manner. The animals were exposed to coarse (CP), fine (CP) and ultrafine particulate (UFP). A control group was exposed to filtered air (FA). The semi-quantitative levels of Angiotensin Receptor Type-1 (*At1r*) and Angiotensin-I Converting Enzyme (*Ace*) mRNA after acute (3 days, 5 h/d) and subchronic (8 weeks, 5 h/d, 4 d/week) exposure are shown. Scatter dot plot shows the value of the median. Below each graph representatives gels illustrating the expression levels of mRNA are shown. * indicates significant differences among groups (*n* = 4 per group, *p* < 0.05)

On the other hand, major and significant mRNA changes were observed for the *At1r* and *Ace* angiotensin system genes in the lungs. These are two of the most important genes in this system and are therapeutic targets in conditions such as hypertension (Fig. 1). For both periods of exposure, we found an increase in the levels of *At1r* mRNA in the groups exposed to FP and UFP compared with those exposed to FA (Fig. 1a and b). In contrast to the effects observed with FP and UFP fractions, we observed a decrease in *At1r* mRNA levels in the group subchronically exposed to CP (Fig. 1b). The *At2r* mRNA levels were not significantly different among any of the types of particle and exposure time conditions as shown in Table 1. Our results for the *Ace* mRNA levels showed a significant increment following the acute exposures to FP and UFP, but not for the subchronic exposures (Fig. 1c and d). On the other hand, the group exposed subchronically to CP responded with a large increase in the *Ace* mRNA relative to all other groups (Fig. 1d). It has been reported that RAAS elements can be expressed constitutively within the cells of various tissues and can have intracrine, paracrine and endocrine effects in the organism [10]. Angiotensin-II is the product of the enzymatic activity of ACE and is the ligand for AT_1_R and AT_2_R. Lung tissue expresses these three genes, which are important in the regulation of the pulmonary circulation. As a result of the exposures, *At1r* and *Ace* responded to PM, but *At2r* did not. This suggests that PM exposure modulates the activation of angiotensin-I and the deactivation of bradykinin by up-regulating *Ace* and promoting *At1r* up-regulation However, the regulation of *At2r,* which has been considered to have an antagonistic effect on the *At1r* signaling pathway, was not affected.

All of these results indicate that PM can regulate two of the principal endocrine systems involved in the regulation of the cardiovascular system at mRNA level. Our data also showed that KKS responds acutely, and not subchronically. On the other hand, the expression of the RAAS genes increased at the end of both exposures, but the responses differed according to the type of particle and the specific genes affected. The mRNA levels of both endocrine systems suggest that KKS and RAAS genes are implicated in the physiological response induced by PM, and these gene expression changes may result in changes in the protein levels.

### Protein levels of AT_1_R and ACE in the lungs

Following the screening of the expression of RAAS- and KKS-associated genes, we investigated whether the protein levels of AT_1_R and ACE, which demonstrated the most significant changes in the mRNA, were also altered.

**Fig. 2:**
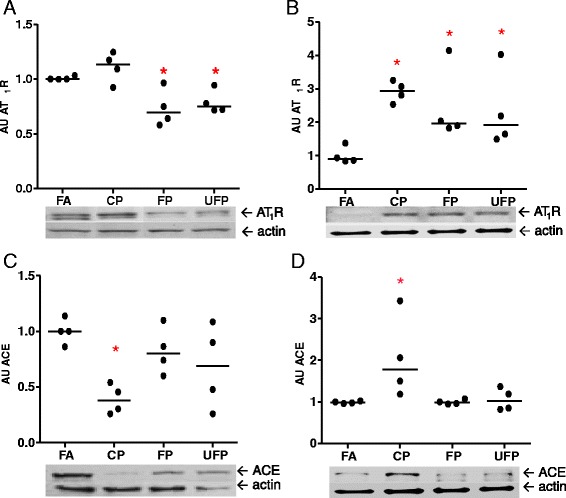
Particle matter exposure regulates AT_1_R and ACE protein in a particle size-dependent manner. Animals were exposed to coarse (CP), fine (FP) and ultrafine particulate (UFP), or filtered air (FA). **a**) and **b**) The protein levels of Angiotensin Receptor Type-1 (AT_1_R) and **c**) and **d**) Angiotensin-I Converting Enzyme (ACE), after acute (3 days, 5 h/day) and subchronic (8 weeks, 5 h/day, 4 days/week) exposure, respectively, are shown as arbitrary units (AU). Scatter dot plot shows the value of the median. * indicates significant differences among groups (*n* = 4 per group, *p* < 0.05)

In the lung total protein extracts, we observed a statistically significant reduction in the levels of the AT_1_R protein after acute exposures to FP or UFP (Fig. 2a). However, after the subchronic exposure, our results showed an increase in the AT_1_R protein levels for all three particle fractions (Fig. 2b). Moreover, we observed a decrease in the ACE protein levels after acute exposure to CP, while in the subchronic exposures, an increase in ACE levels was observed. However, for both acute and subchronic exposures to FP and UFP the ACE protein levels were variable. Therefore, although we observed a decrease in the protein levels, the differences were not statistically significant (Fig. 2c and d).

Our results show that exposure to PM was able to induce the ACE and AT_1_R proteins as well as mRNA. Differences were observed with respect to the particle fractions and the target proteins. Acute exposure to CP caused a down-regulation of the ACE protein, without a change of the AT_1_R protein levels. In contrast, the subchronic exposure to CP induced an increase in ACE protein, which was accompanied by the up-regulation of AT_1_R. However, FP and UFP did not affect the ACE protein levels. Instead, AT_1_R was the main molecular target of the FP and UFP fractions. No differences between the FP- and UFP-induced effects on the AT_1_R protein levels were observed. This could be explained either by the fact that UFP are contained within the FP fraction, which precludes separation of their effects, or because they can reach the same region in the lungs, they induce similar responses.

Our data suggest that PM can regulate AT_1_R and ACE protein levels in the lungs in a time- and possibly a PM size-dependent manner. It is important to highlight that the CP, for which we did not expect a response, was able to modulate both genes, while the FP and UFP fractions only modulated AT_1_R. This evidence confirms that CP can promote pulmonary events as well as the PM smaller than 2.5 μm, and suggests a possible contribution to cardiovascular impairment as a consequence of exposure to PM.

### [^3^H]-Angiotensin-II binding to lung membranes

**Fig. 3:**
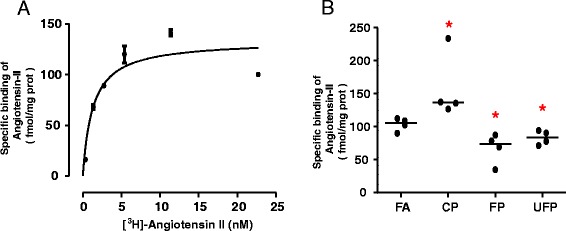
Acute exposure to PM modifies [^3^H]-Angiotensin-II binding to the lung tissue membranes. **a**) Saturation binding. Membranes, obtained from naïve animals as described in Methods, were incubated with the indicated concentrations of [^3^H]-Angiotensin-II. Specific receptor binding was determined by subtracting the binding in the presence of 100 μM telmisartan from the total binding. The points show the means from quadruplicate determinations from a single experiment, which was repeated twice more with membranes obtained from different naïve animals. The line drawn is the best fit to a hyperbola. Best-fit values for the equilibrium dissociation constant (*K*_d_) and maximum binding (B_max_) are given in the text. **b**) Single-point determinations. Membranes were obtained from animals exposed to coarse (CP), fine (FP) and ultrafine particulate (UFP) or from the filtered air control group (FA), and then incubated with 10 nM [^3^H]-Angiotensin-II. Specific receptor binding was determined by subtracting the binding in the presence of 10 μM telmisartan. Scatter dot plot shows the value of the median. * Indicates significant differences among groups (*n* = 4 per group, *p* < 0.05)

Similar to other G protein-coupled receptors, upon activation AT_1_Rs can be internalized from the cell membrane to the cytoplasm and then can be either recycled or degraded. Because the reduction in AT_1_R protein after the acute exposure to FP and UFP did not match the mRNA levels, we performed a binding assay with labeled Ang-II as an alternative method to confirm the protein levels. First, we obtained a saturation curve to determine the receptor density and the affinity for [^3^H]-Angiotensin-II in lung membranes obtained from rats that were not used in the PM experiments. These determinations (Fig. 3a) yielded a maximum specific binding (B_max_) of 90 ± 10 fmol/mg protein (3 experiments) and an equilibrium dissociation constant (*K*_d_) of 2.8 nM (p*K*_d_ 17.2 ± 7.7).

In single-point determinations with a near saturating concentration (10 nM) of [^3^H]-Angiotensin-II, a reduction in the specific binding was observed in lung membranes from the animals exposed to FP and UFP. In contrast, exposure to CP increased the specific [^3^H]-Angiotensin-II binding (Fig. 3b).

The binding data thus showed the same pattern as the total protein detected immunologically. On this basis, we infer that the internalization and degradation of AT_1_R in the acute exposure could be induced by different mechanisms such as an over-activation, a target of oxidative stress and/or allosteric regulation by divalent cations, similar to observations of other GPCRs [45, 46].

### AT_1_R levels in subcellular fractions of lung tissue

**Fig. 4:**
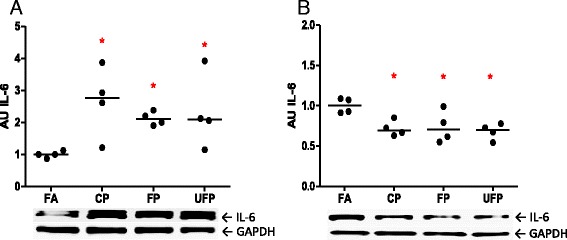
Particle matter modifies the AT_1_R subcellular distribution in lung tissue in a time-dependent manner. Representative gels of Angiotensin-II type-I receptor (AT_1_R) detection in lung nuclear and non-nuclear fractions after acute (upper panel) and subchronic (lower panel) exposure to coarse particulate (CP), fine particulate (FP), ultrafine particulate (UFP) or filtered air (AF). We used GAPDH and acetylated Histone-4 (H_4_ac) as cytosolic and nuclear quality control targets, respectively, and actin was used as a general protein loading control. Representative blot of *n* = 4

The conventional paradigm for GPCRs indicates that the activated receptors are internalized from the plasma membrane to the cytoplasm where they may be degraded or recycled [47]. However, new evidence indicates that some GPCRs can be localized in the nuclear envelope [48]. In the case of AT_1_R, Lu et al. reported that after 15 min of stimulation of neuronal cell cultures with Ang-II, AT_1_R was sequestered to the nuclear membrane [49]. However, Tadevosyan et al. [50] demonstrated that the presence of angiotensin receptors (AT_1_R and AT_2_R) in the nuclear membrane is most likely a result of intracellular synthesis and trafficking, and not the result of post-endocytotic trafficking in cardiomyocyte cultures. For these reasons, we evaluated the distribution of AT_1_R protein between the nuclei and the rest of the cell in the lung tissue following exposure to airborne PM in both exposure schemes (Fig. 4). For the acute exposures, we observed an increase in AT_1_R in the non-nuclear fraction in all exposed groups, while in the nuclear fraction we observed a reduction in protein levels (Fig. 4, upper panel). On the other hand, following the subchronic exposures, we observed the opposite response: under these conditions, we observed a decrease in AT_1_R levels in the non-nuclear fraction and an increase in this protein in the nuclear fraction (Fig. 4, lower panel).

On the basis of our findings with respect to the subcellular distribution of AT_1_R in the lung tissue, we suggest that this receptor may mediate processes that include cell proliferation, survival, inflammatory responses, DNA synthesis and transcription as has been demonstrated for other GPCRs found in the nuclear envelope [48]. For example, nuclear AT_1_R activation has been demonstrated using isolated nuclei from fetal rat myocytes. Stimulation of these nuclei with angiotensin-II induced transcription as indicated by [α-^32^P] UTP incorporation, and this effect was inhibited by pertussis toxin [50].

### IL-6 cytokine protein levels in lung

**Fig. 5:**
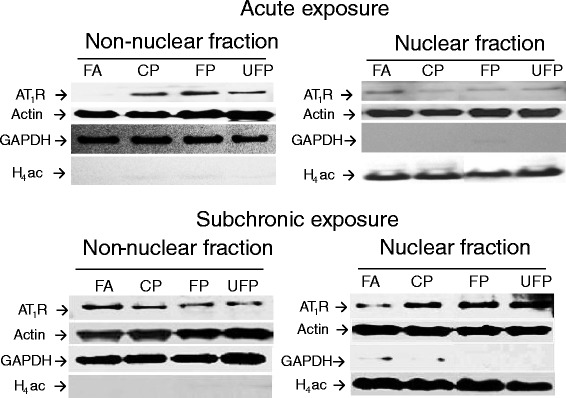
Acute, but not subchronic, exposure to particle matter increases IL-6 in lungs. Animals were exposed to coarse (CP), fine (FP) and ultrafine particulate (UFP), or filtered air (FA). The protein interleukine-6 (IL-6) levels after **a**) acute (3 days, 5 h/day) and **b**) subchronic exposure (8 weeks, 5 h/day, 4 days/week), are shown as arbitrary units (AU). Scatter dot plot shows the value of the median. * indicates significant differences among groups (*n* = 4 per group, *p* < 0.05)

Pleiotropic cytokine IL-6 is involved in the acute phase of inflammation and accelerates coagulation induced by PM exposure *in vivo* [5]. In addition, in endothelium cells, IL-6 induced the expression of AT_1_R [24]. We observed a statistically significant increase in lung IL-6 protein from the acute exposure to the three PM fractions (Fig. 5a); on the other hand, in the subchronic exposure a down regulation in the three PM groups below the levels observed in the FA group was observed (Fig. 5b). Our results indicate the induction of lung IL-6 as a marker of the acute phase of the inflammation process. IL-6 could be involved in the up-regulation of AT_1_R in lung acute PM exposure. In contrast, in the subchronic exposure a lack of induction of lung IL-6 protein was observed and AT_1_R expression was increased. If a molecular communication process takes place between IL-6 and AT_1_R it only happens acutely, but not subchronically, yet this hypothesis needs to be confirmed.

### Expression of angiotensin and bradykinin system genes in the heart

The results obtained in the lungs suggest a contribution of the RAAS and to a lower degree, but not less important, of the KKS in the endocrine pulmonary response to PM damage. These heterogeneous responses of gene expression observed in the lungs following the acute and subchronic exposures to airborne particulate fractions could be conveyed from the lungs to the heart.

As expected, we observed a response in the mRNA levels of bradykinin-related genes in the heart following the exposure to PM. Similar to the observations in the lungs, in the heart there was an increase in *Klk-1* after the acute exposure to FP that was sustained in the subchronic exposure. Additionally, we observed an increase in *B1r* in all groups acutely exposed to particulate (Table 2). We were not able to detect *B2r* in the heart samples; no positive amplification was observed in the heart samples from any of the exposure schemes. This observation confirms that, as previously reported, *B2r* is a receptor which has a null or low expression in the heart ventricles of the adult male rat [51–53].

**Table 2:** Particulate matter effects on gene expression of angiotensin and bradykinin systems in the heart. Semi-quantitative expression results for angiotensin-receptor type-2 (At2r), Bradykinin receptor type-1 (B1r) and kallikrein (Klk-1) enzyme after acute and subchronic exposures

Acute exposure				
Group	FA	CP	FP	UFP
Gene				
*At2r*	1	1	1	1
	(0.8-1.2)	(1-1.1)	(0.9-1.1)	(0.5-1)
*Ace*	1	1.6 *	1.1	1.7 *
	(0.9-1.1)	(1.4-1.8)	(0.9-1.3)	(1.1-2.1)
*B1r*	1	1.7 *	2.2 *	1.9 *
	(0.8-1.3)	(1.4-2.2)	(2.1-2.6)	(1.3-2.4)
*Klk-1*	1	1.2	1.6 *	1.4 *
	(0.8-1.2)	(1-1.5)	(1.4-1.9)	(1.2-1.5)
Subchronic exposure				
Group	FA	CP	FP	UFP
Gene				
*At2r*	1	0.97	0.92	0.71 *
	(0.96-1.03)	(0.97-1.05)	(0.72-0.9)	(0.7-0.8)
*Ace*	1	1	1	0.9
	(0.9-1.1)	(0.9-1.1)	(0.9-1.3)	(0.8-1)
*B1r*	Not determined			
*Klk-1*	1	1.1	1.2 *	1.1
	(0.9-1.1)	(0.9-1.2)	(1.19-1.22)	(1-1.3)

Acute exposure was defined as 5 h per day for 3 days

Subchronic exposure was defined as 5 h per day, 4 days per week, during 8 weeks

All results were corrected with glyceraldehyde 3-phosphate dehydrogenase as a housekeeping gene

AF: Air filtered

CP: Coarse particulate

FP: Fine particulate

UFP: Ultrafine particulate

Data are showed as median and range

* Indicates significantly different from FA

Our results for the expression of KKS genes show that there is a differential response between the lungs and the heart: the heart responded to the exposures to the three particulate with the induction of *B1r* mRNA. These results suggest that the inflammatory factors or chemical components are released from the lungs and enter in the blood stream of the pulmonary circulation before being translocate to the heart. Although we observed this response to all three particulate, FP, but not UFP, was able to induce *Klk-1,* which indicates that there are differences between these two particle fractions and suggests that FP could affect the metabolism of kinin peptides.

**Fig. 6:**
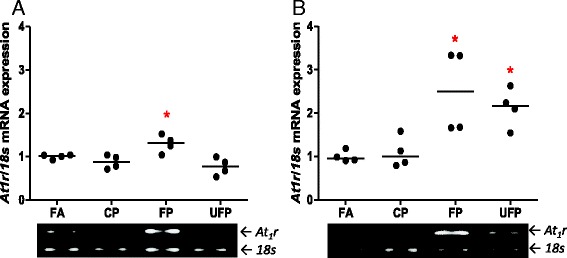
Acute and subchronic exposures to fine and ultrafine PM up-regulate heart AT_1_R mRNA. The animals were acutely (5 h/day, 3 days) or subchronically (5 h/day, 4 days/week, 8 weeks) exposed to coarse (CP), fine (FP) and ultrafine particulate (UFP) or to filtered air (FA). Semi-quantitative levels of Angiotensin Receptor type-1 (*At1r*) mRNA after **a**) acute and **b**) subchronic exposures in the heart. Scatter dot plot shows the value of the median. * indicates significant differences among groups (*n* = 4 per group, *p* < 0.05)

With respect to the angiotensin system, the heart *At2r* mRNA levels were not significantly different among the acute exposure groups. In contrast, subchronic exposure to UFP caused a significant decrease in the heart *At2r* levels (Table 2). The Ace mRNA increased after acute exposure to either CP or UFP, but these changes did not persist in the subchronic exposure (Table 2). We observed an increase in the *At1r* mRNA levels after acute and subchronic exposure to FP, as well as in the group after the subchronic exposure to UFP (Fig. 6).

The cardiac over-expression of AT_1_R has been demonstrated to be involved with the promotion and exacerbation of myocardial impairment. It is relevant to note that short PM exposure was able to induce *At1r*, which was maintained in the subchronic exposure. This observation was accompanied with a down-regulation of *At2r* only after the subchronic exposure, indicating that exposure to PM, an environmental air pollutant, can increment the expression of *At1r* in the absence of counterbalancing changes in the expression of *At2r*.

### Up-regulation of *Col3a1* and *Acta1* in presence of AT_1_R and IL-6 in the heart

**Fig. 7:**
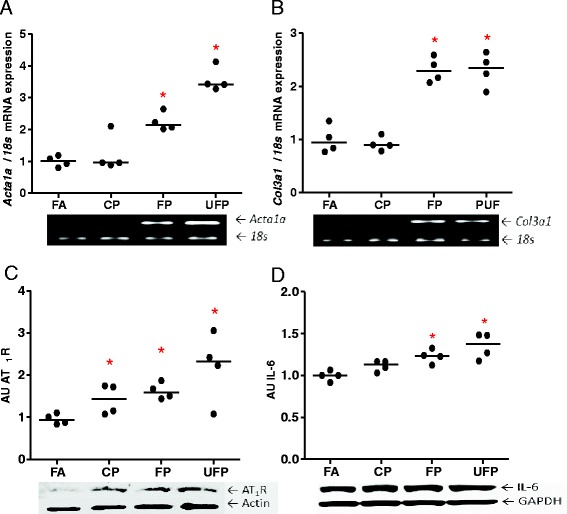
Subchronic exposure to fine and ultrafine PM induces heart reprogramming and up-regulates AT_1_R and IL-6. The animals were subchronically exposed (5 h/day, 4 days/week for 8 weeks) to coarse (CP), fine (FP) and ultrafine particulate (UFP) or to filtered air (FA). **a**) Semi-quantitative levels of alpha-skeletal actin (*Acta1a*) mRNA and **b**) Semi-quantitative levels of collagen-III (*Col3a1*) mRNA in the heart. Protein levels of AT_1_R and interleukin-6 (IL-6) are expressed in arbitrary units (AU), **c**) and **d**), respectively. Scatter dot plot shows the value of the median. * indicates significant differences among groups (*n* = 4 per group, *p* < 0.05)

Two of the main classes of response genes in the heart during pathological states of cardiac damage are the heart fetal reprogramming genes and the expression of genes involved in the deposition of the extracellular matrix. We chose alpha skeletal actin (*Acta1*) and collagen-III (*Col3a1*) as two representative markers of myocardial adaptive response to damage. We analyzed these genes in heart ventricular samples after the acute exposures, and did not observe any response in either gene (data not show). In contrast, the subchronic exposures to FP and UFP induced the up-regulation of *Acta1* and *Col3a1* (Fig. 7a and b).

Our results indicate that PM is not able to acutely up-regulate these genes, but they are increased by subchronic exposure. The presence of these two markers in various rat cardiac disease models has been described, i.e., hypertension or induced cardiac infarction [54]. FP and UFP have been associated predominantly with the enhancement of the development of cardiovascular diseases. Our results confirm that particulate less than 2.5 μm are able to up-regulate gene reprogramming in the heart, possibly by through an increase in blood pressure.

There is substantial evidence to support the concept that RAAS induces heart gene reprogramming [54–57]. For this reason, we evaluated AT_1_R total protein levels in samples from the subchronic exposure where we observed heart gene reprogramming. We also observed an increase in the levels of AT_1_R in the total protein that seems PM size-dependent, where the highest increase was observed for UFP followed by FP and to a lower degree for CP (Fig. 7c).

IL-6 cytokine is involved in the induction of AT_1_R in the endothelium; also Ang-II through AT_1_R induces the expression of cardiotrophin-1, leukemia inhibitory factor and IL-6, all which are members of IL-6 family. In rodent cardiac fibroblast these cytokines induce cardiomyocyte hypertrophy through the activation of gp130, which is a IL-6 receptor [58]. In this sense, we evaluated IL-6 protein levels, as a marker of inflammation response, in the heart and observed an increment in IL-6 that could be size-dependent where FP and UFP showed statistical significant differences (Fig. 7d) and they have the same tendency as the AT_1_R in the heart. Our findings suggest that IL-6 and AT_1_R are induced in heart by PM exposure and is possibly size particulate-dependent; also, IL-6 and AT_1_R show the same behavior as cardiac reprogramming genes induced by FP and UFP.

### Histology of intramyocardial coronary arteries

**Fig. 8:**
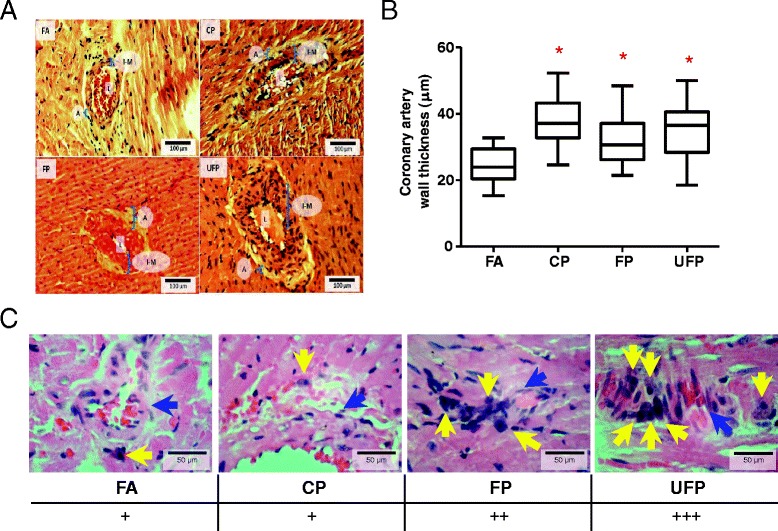
Subchronic PM exposure increases intramyocardial coronary artery wall thickness independently of PM size. Hematoxylin & Eosin staining of 5 μm myocardial slices. **a**) Representative slides of the hearts from rats exposed to Filtered Air (FA), coarse (CP), fine (FP) and ultrafine (UFP) particulates for 5 h/day, 4 days/week for 8 weeks. **b**) Box plot of ten random measurements of each coronary artery wall measured in the tissues. **c**) Semi-quantitative analysis of the presence of mononuclear cells in heart coronary arteries in the groups exposed to FP and UFP. L: Artery lumen; A: Tunica adventitia; I-M: Tunica intima-media. Yellow arrows indicate mononuclear cells and blue arrows indicate a blood vessel. Scatter dot plot shows the value of the median. * indicates significant differences among groups (*n* = 4 per group, *p* < 0.05)

One effect of RAAS in the heart tissue is hypertrophy and proliferation of the smooth muscle cells of the blood vessels. We evaluated the thickness of the intramyocardial coronary arteries (we considered only arteries found within the myocardium wall, but not in the epicardium or endocardium) as a morphological effect in myocardial tissue induced from the particle exposure and found that the exposure to the three particle fractions increased the intramyocardial coronary artery wall thickness (Fig. 8a and c). This result is consistent with the induction of AT_1_R with the three fractions and suggests the activation of RAAS.

It has been proposed that particulate less than 2.5 μm can pass through the lung respiratory and vascular walls and reach the heart through the pulmonary circulation [48, 59]. A particular observation in this study was the presence of mononuclear cells, which seem to be macrophages as they display fried egg-like morphology, next to the arteries in the heart from the UFP group and to a minor degree from the FP group (Fig. 8c). This observation suggests an immune cellular response at the end of the subchronic exposure to FP and UFP.

### Lungs and heart subchronic antioxidant response

**Fig. 9:**
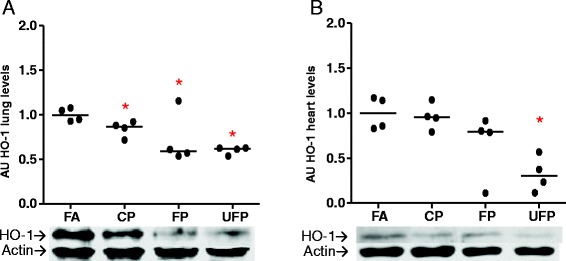
Particle size-dependent decreases in heme oxygenase-1 after subchronic exposure in the lungs and heart. Animals were exposed subchronic to coarse (CP), fine (FP) and ultrafine particulate (UFP) or to filtered air as a control (FA) for 8 weeks (5 h/day, 4 days/week). Protein levels of heme oxygenase-1 (HO-1) in arbitrary units (AU) in lungs **a**) and heart **b**). Scatter dot plot shows the value of the median. * indicates significant differences among groups (*n* = 4 per group, *p* < 0.05)

Finally, one of the most important responses to particulate exposure is the antioxidant defense. We evaluated the protein levels of γ-glutamyl-cysteine-synthetase catalytic subunit (γGCSc), and heme oxygenase type-1 (HO-1) in the lungs and heart after subchronic exposure to airborne PM. We did not observe significant changes in γGCSc protein levels in either tissue (data not shown). However, the lung levels of HO-1 seem to decrease in a particulate size-dependent manner, where the greatest down-regulation was observed in the UFP group (Fig. 9a). The same tendency was observed for the heart levels of HO-1, but only the effects of UFP were statistically significant (Fig. 9b).

We did not observe differences in γGCSc protein levels in lungs and heart even though glutathione is one of the first antioxidant defenses against oxidative stress. Also, although γGCSc is involved in the *de novo* synthesis of glutathione the toxic effect of PM may be directly modifying the reduced and oxidized glutathione ratio and diminish total glutathione levels, as it has been reported for different progressive lung diseases [60]. Also, other antioxidant response elements such as ascorbic acid, which is abundant in the rat lung, or HO-1 and bilirubin in the cardiovascular system, could be involved in the response against PM-induced oxidative toxicity [61].

HO-1 is an inducible stress protein enzyme that has major role in cytoprotective defense against apoptosis, inflammation and oxidative stress through the catabolism of the heme moiety and the release of Fe^2+^, biliverdin and CO. The latter two metabolites are implicated in the antioxidant response and vascular tone, respectively [62–64]. Low HO-1 levels have also been shown to be associated with an impairment of the cardiovascular system [65] and the development of lung diseases [66]. In addition, HO-1 levels can be down-regulated at the mRNA and protein levels by angiotensin-II through AT_1_R in rat vascular smooth muscle cells [67]. Evidence from *in vitro* models (RAW 264.7, THP-1, BEAS-2B and A549 cells) exposed to PM indicated that the induction of HO-1 depended on the PAH content of the PM [68–70]. However, exposure of A549 cells to 9,10-phenanthraquinone down-regulated the expression of HO-1 [71]. Acute *in vivo* studies demonstrate increases in HO-1 protein and mRNA. Nevertheless, in older current smokers, HO-1 mRNA is down-regulated [72]. Furthermore, it has been observed in human populations that a larger size of the dinucleotide repeat polymorphism (GT) n in the HO-1 gene confers a low induction of mRNA by tobacco smoke and is related to the development of emphysema [73]. The same polymorphism has been associated with a low induction and the development of coronary artery disease [74]. Moreover, oxidative stress during the subchronic exposure to PM is likely to be neutralized by non-enzymatic antioxidants derived from enzymatic antioxidant elements, such as heme-metabolism/HO-1/bilirubin-biliverdin biochemical route, these products, bilirubin and biliverdin, play the main antioxidant role, thus the induction of HO-1 is probably not the primarily antioxidant biomarker in response to the subchronic exposure.

### General overview

PM pollution contributes to the development of cardiovascular diseases. Our results indicate that in addition to inflammation and oxidative stress, exposure to PM induces an endocrine response through the expression of some RAAS and KKS elements. This effect could be dependent on the particle size, the tissue type and the time of exposure, and could participate in cardiovascular events.

The KKS acute exposure response was characterized by the induction of the *Klk-1* in the lungs and heart of the groups exposed to FP and UFP. Expression of *Klk-1* leads to the generation of kinin peptides through the breakdown of KNG in tissues. However, this enzyme also can have other substrates such as pro-renin, pro-insulin, LDL, the precursor of atrial natriuretic factor, pro-collagenase, vasoactive intestinal peptide and ANG. Despite the many substrates of KLK-1, it has been reported that KLK-1 improves cardiac physiology reducing blood pressure, prevents the increment in heart mass, reduces the septal thickness and cardiomyocyte size in hypertrophic animal models after aortic constriction. Also, in these animals KLK-1 contributes to decrease the NADPH oxidase activity and increases NO production through B_2_R [75]. Moreover, *klk-1* null mice do not show alteration in blood pressure but exhibited abnormal structures (thin wall and diminished mass of the left ventricle) and functions (reduced cardiac output, stroke volume and left ventricular fractional shortening) of the heart [76].

The above evidence indicates the importance of KLK-1 in the physiology of the cardiovascular system, in addition, kallikrein family genes have been proposed as emerging new markers for the development of cancer [77], and have been weakly associated with the development of lung diseases. It has been demonstrated that KLK-1 is abundant in the lung lavage from asthmatic patients challenged with allergen [78], as well as in an allergic sheep model. Furthermore, a monoclonal antibody to KLK1 inhibited allergen-induced late-phase bronchoconstriction and airway hyper-responsiveness to carbachol. Additionally, in human tracheobronchial epithelial cells, this antibody blocked oxidative stress-induced epidermal growth factor receptor activation and mucus hypersecretion [79].

This evidence suggests that in the lung KLK-1 seems to be involved in the triggering of local inflammatory response, through breakage of KNG zymogen to bradykinin and kallidin by KLK-1. Bradykinin and kallidin are further broken by the membrane-bound carboxypeptidase-M into des-Arg-bradykinin and des-Arg-kallidin peptides, which activates B1R that is an important inflammatory mediator [8].

For these reasons, the induction of *Klk1* by FP and UFP suggests that the generation of kinin peptides or other substrates of KLK1 could be modified by the PM-induced activation.

The bradykinin receptors did not show a response in the lungs at any time of exposure. However, in the heart, the expression of *B1r* increased after the acute exposure in the three PM-exposed groups. *B1r* can be induced by inflammatory cytokines, and it can regulate the induction of iNOS enzyme. Thus, our results indicate that the three fractions of particulate could induce the release of secondary mediators from the lungs to the heart, where they were able to induce *B1r*.

With respect to RAAS, we observed that *At2r* did not change significantly at any exposure times in the lungs. In the heart, we only observed a decrease in the mRNA for this receptor after the subchronic exposure to UFP. The lack of response of *At2r* indicates that PM mainly promotes *At1r* expression more than the other mRNAs.

*Ace* is of particular interest because it can be a target of PM toxicity due to having a zinc prosthetic group that could be replaced by other divalent cations. Furthermore, this enzyme can activate angiotensin and degrade bradykinin. We observed that the ACE mRNA and protein in the lungs were regulated mainly by CP, and not by FP and UFP, most likely because CP can only reach the bronchial branches and does not penetrate the lungs as deeply as FP and UFP. Future studies should be conducted to determine whether ACE protein is degraded or is released from the lungs to the blood stream after acute exposure to PM. This is important given that new evidence indicates a novel pathway in which activated ACE can be released from the cell membrane [80–83].

The principal finding of our work was that PM regulates AT_1_R after acute and subchronic exposure. Although CP did not induce the mRNA for AT_1_R, it was able to increase the protein levels of this receptor as observed using the binding assay and Western blotting. The potential mechanism for the increase in AT_1_R protein without the mRNA up-regulation in the CP group, it could be explained as reported by Mori et al. [84], cis-acting sequences in the AT_1_R gene and the glucocorticoid-responsiveness elements control the transduction process, however, AUG codon in the 5’-leader of AT_1_R transcripts play a role in the translation regulation of AT_1_R protein, future research is necessary to confirm this hypothesis. These results confirmed that CP may contribute in the development or promotion of lung and heart diseases, through the increase of Ang-II binding and the regulation of ACE.

With respect to FP and UFP, we know that the UFP fraction is included within the FP fraction. For some parameters that we evaluated, such as mRNA and protein levels, both fractions induced the same degree of response in the lungs. However, we were able to distinguish different responses between the fractions in the heart expression of *Acta1*, HO-1 and AT_1_R proteins. Thus, differences between FP and UFP can be attributed to the different composition and the deposition sites in the respiratory tract reached by these two different particulate types.

Particle chemical composition has an important role in particle-induced toxicity because it can promote oxidative stress through chemical catalysis or metabolism and can enhance inflammatory responses. Previously, the chemical composition of PM_10_ and PM_2.5_, including the metal and organic partial composition was reported by Guerra et al. [85]. The total metal mass concentrations of the CP and FP were 5488.89 and 2992.11 ng/m^3^, respectively. We estimated the percent of each metal mass contribution from the total metal mass of CP and FP based on the results from Guerra et al. [85] (Table 3). For ten metals (Fe, Zn, Pb, Mn, Cr, Cu, Ni, V, Co and Cd), we found that the element with greatest mass contribution to CP and FP was Fe, at 38.5 and 48.4 %, respectively. The second most abundant element contributing to the CP and FP masses was Zn, at 37.9 and 12.1 %, respectively. The rest of the metals had a minor contributions to particle mass (less than 12 %), but some elements are more abundant in FP than CP. For example we found more Cd in FP than CP (44.6 times), followed by Co (6.7 times); V (5.1 times); Cu (2.6 times); Mn (2.2 times) and Pb (1.2 times). Biologically relevant effects of the particle composition on RAAS were reported by Li et al. [42], who demonstrated that particle constituents such as V and Cu induce pulmonary artery vasoconstriction through AT_1_R and ACE. According to the authors, other metals including Ni, Fe, Mn, Zn and Al produced a weak or no effect on vasoconstriction. Based on the percent contribution to the mass, Zn was one of the most abundant. It was shown that Zn contained in PM inhibits total cardiac aconitase activity, induces mitochondrial DNA damage, and causes modest changes in the cardiac expression of mRNAs involved in signaling, ion channel function, oxidative stress, mitochondrial fatty acid metabolism and cell cycle regulation [86].

**Table 3:** Metal mass contribution in coarse (CP) and fine PM (FP) from subchronic exposure

Metal	CP %	FP %
Fe	38.5	48.4
Zn	37.9	12.1
Mn	5.0	11.1
Pb	6.9	8.3
Cu	4.0	10.3
Cr	4.8	1.7
V	1.0	5.0
Co	0.2	1.2
Cd	0.03	1.2
Ni	1.7	0.7

On the basis of the information presented above and considering the metal composition of the PM in our study, we suggest that the biological effects observed could be related to the presence of divalent metals that are able to mimic prosthetic groups, such as Zn in the ACE enzyme, or mediate oxidative stress and promote inflammation and thus up-regulate AT_1_R. Although CP and FP share some components, we suggest that the biological effect attributed to PM-associated metal content is related to the sites in the lungs reached by the particulate and their potential to translocate beyond the lungs.

In this study it was not possible to assess UFP mass and collect it for chemical determination. However, evidence in the literature [87–89] allows to propose the participation of divalent metals (i.e., Zn, Cu, Cd, etc.) as the UFP-chemical components mostly involved in the induction of the effect on RAAS and KKS. Moreover, we do not discard that some other components (i.e., endotoxin and possibly other metals) may also contribute to induce this effect as a secondary response of the release of inflammatory mediators.

While particle composition and possibly oxidative stress could explain the expression of AT_1_R, the second most important mechanism of toxicity from the exposure to PM is the inflammatory process. Two cytokines associated with AT_1_R expression are the pleiotropic cytokine IL-6 [24, 90] and growth factor TGF-β [91, 92], both of which are also involved in the pathological states of lung and heart disease. We evaluated IL-6 levels as an inflammation marker as well as an AT_1_R inductor. We observed that an inflammation response was present in our experimental model in the lungs and heart. These data suggest that IL-6 may participate in the regulation of AT_1_R in the acute phase of the PM exposure but not in the subchronic exposure in the lung. We suggest that in the subchronic exposure the RAAS by itself maintains the induction of AT_1_R, or inflammatory and/or oxidative stress responses in the lungs may also be involved. On the other hand, heart IL-6 levels in the subchronic exposure seem to increment in a size-dependent manner as well as we observed in the heart AT_1_R protein. However, future studies should focus to establish whether IL-6 is determinant in the induction of AT_1_R in the heart since it has been reported that cytokines such as TNFα [23] and IL-1α [25] are involved in the induction of AT_1_R in rat cardiac fibroblast and smooth muscle cells, respectively.

It is important to highlight that the differences in the PM concentration achieved in the chambers could directly influence the biological responses observed in the present study. The chamber PM concentrations were ~32 μg/m^3^ for CP, ~178 μg/m^3^ for FP, and ~107 μg/m^3^ for UFP. These concentrations suggest the possibility that the highest biological responses should be observed in PM concentration-response relationship, nevertheless, our data seems to indicate that the molecular targets (e.g., AT_1_R, HO-1 etc.) respond in a particulate size-dependent manner. However, some biological responses were independent of the concentration or the particle size such as the *B1r* expression in heart in the acute exposure, lung IL-6 levels or the intramyocardial thickness, and could be influenced by PM composition, yet this hypothesis needs to be confirmed. Moreover, we have to consider that our results, based in our experimental design, have uncertainty from other factors that include the detailed PM chemical content, the retained dose and the intrinsic biological background.

Future directions of our study should focus in AT_1_R independent ligand-activation since it has been reported that AT_1_R can function as a mechanoreceptor that under mechanical stretch *in vitro* may induce RAAS elements [93, 94], and probably the physical interaction of PM with AT_1_R in the cellular membrane may influence the activation of AT_1_R pathway.

Finally, our results demonstrate that the three fractions of PM were able to modulate their effects through the deregulation of two endocrine systems involved in pulmonary and cardiovascular pathology. These effects could result from the development of the inflammation and oxidative stress processes feeding back on the response of the RAAS.

In this sense our results suggest that the cardiac effect of PM is consequent to a neuro-endocrine stimulation, which includes RAAS and KKS response, probably by mediating vascular tone dysfunction (i.e., hypertension) in the lung as well as in the heart. The augmented AT_1_R expression observed in this study in the lungs and heart may be related with both processes.

## Conclusion

Exposure to PM is detrimental to human health. Oxidative stress and inflammation are the major mechanisms that contribute to the impairment of the lung and cardiovascular systems. Furthermore, in the present study we observed that PM induces the expression of the endocrine system RAAS and KKS elements in a time- and probably in a particulate size-dependent manner. PM primarily promotes the expression of AT_1_R in the lungs and heart, which appears to be involved in the depletion of the antioxidant HO-1, as well as the tissue remodeling and heart gene reprogramming resulting from a subchronic exposure to FP and UFP, concomitantly, PM induces acute IL-6 response in the lung and subchronic response in the heart. Thus, these findings contribute to the understanding of the underlying mechanisms involved in the development of cardiac disease associated with PM exposure.

## Methods

### Inhalation exposure

The present study was carried out in the north of Mexico City, an area with high traffic affluence and industrial activity [95], during the months of May to July, 2009. The particulate concentrator was localized within the Animal Care Unit at CINVESTAV-IPN.

Male Sprague Dawley rats (purchased from Harlan, Mexico; 4 rats/group) were exposed acutely (3 days, 5 h per day) and subchronically (8 weeks, 4 days/week, 5 h per day) to coarse (CP), fine (FP), or ultrafine (UFP) particulate using an aerosol enrichment concentrator system. A control group was exposed to filtered air (FA). The FP fraction was defined as particulate less than or equal to 2.5 μm, which includes the UFP fraction. The particulate concentration in the exposure chambers was 32, 178 and 107 μg/m^3^ for CP, FP, and UFP, respectively. Simultaneous air ambient concentration of PM_10_ and PM_2.5_ were monitored and those results were accompanied with the partial chemical composition of PM_10_ and PM_2.5_ in the study developed and reported by Guerra-García et al. [85].

At the end of the each exposure (24 h after the last exposure), the animals were anesthetized (i.p. 10-20 mg/kg of sodium pentobarbital) and euthanized by exsanguination. The lung and heart tissues were dissected and frozen in liquid nitrogen and stored at -70 °C until analysis. A ventricular slice of the heart was fixed in 10 % buffered formaldehyde and embedded in paraffin for histological analysis.

### Semi-quantitative reverse transcription-polymerase chain reaction

Total RNA was isolated from the lungs and heart using TRIzol reagent (Invitrogen™, Life Technologies, Thermo Fisher Scientific, Carlsbad, CA, USA). cDNA synthesis was performed with 3 μg of total RNA according to the manufacturer’s instructions (SuperScript II, Invitrogen™, Life Technologies, Thermo Fisher Scientific, Carlsbad, CA, USA). Specific oligonucleotides were designed for each gene (Table 4) using GeneRunner software, and we confirmed their amplification using Primer-BLAST [http://www.ncbi.nlm.nih.gov/tools/primer-blast/]. We chose three RAAS genes (*At1r*, *At2r* and *Ace*) and three KKS genes (*B1r*, *B2r* and *Klk-1*) on the basis of their roles as the effectors and mediators of the metabolism of the active peptides. To evaluate heart gene reprogramming, we evaluated the mRNA levels of *Acta1* and *Col3a1*. We used *18S* and *Gapdh* as housekeeping genes. After PCR amplification, the PCR products were loaded and subjected to electrophoresis in 1.5 % agarose gels, stained with ethidium bromide and photodocumented in a transilluminator (UVP EC3 imaging system, UVP Inc., Upland, CA, USA). Densitometry was performed with the software Image J (National Institutes of Health, USA).

**Table 4:** Oligonucleotides used for PCR amplification. The gene name, the sequence of forward (Fw) and reverse (Rv) oligonucleotide, the PCR product and the Genbank ID are shown

Gen	Olinucleotides	PCR product (pb)	Genbank ID
*At1r*	Fw 5'-AATATTTGGAAACAGCTTGGT-3'	331	[GenBank: NM_030985]
	Rv 5'-ATGATGATGCAGGTGACTTTG-3'		
*At2r*	Fw 5'-TAGTTCCCCTTGTTTGGTG-3'	428	[GenBank: NM_012494]
	Rv 5'-GAGGATGGCAAAAGGAAGT-3'		
*Ace*	Fw 5'-CCAACAAGACTGCCACCTG-3'	457	[GenBank: NM_012544]
	Rv 5'-GTACTGGTGACATCGAGGTTG-3'		
*B1r*	Fw 5'-AGCATCTTCCTGGTGGTGG-3'	420	[GenBank: NM_030851]
	Rv 5'-CCAGCAGACCAGGAAGGAG-3'		
*B2r*	Fw 5'-GAGATCTACCTGGGCAACCT-3'	599	[GenBank: NM_173100]
	Rv 5'-AGGAAGGTGCTGATCTGGAA-3'		
*Klk-1*	Fw 5'-CCCTCACCCTGACTTCAAC-3'	236	[GenBank: 001005382]
	Rv 5'-TCACACACTGGAGCTCATC-3'		
*Acta1*	Fw 5'-ACATCGACATCAGGAAGGAC-3'	234	[GenBank: NM_019212]
	Rv 5'-CGTCGTACTCCTGCTTGGT-3'		
*Col3a1*	Fw 5'-AGGGTGATCGTGGTGAAAA-3'	239	[GenBank: NM_032085]
	Rv 5'-TCCTCGATGTCCTTTGATG-3'		
*18S*	Fw 5'-GCAGCTAGGAATAATGGAATA-3'	188	[GenBank: NR_046237]
	Rv 5'-GACTTTCGTTCTTGATTAATGA-3'		
*Gapdh*	Fw 5'-ACCACAGTCCATGCCATCAC-3'	166	[GenBank: NM_017008]
	Rv 5'-TGCCAGTGAGCTTCCCGTT-3'		

### Western blot

To obtain the total protein, the lung samples were homogenized with Nonidet-P40 buffer (150 mM NaCl, 1 % NP40, 50 mM Tris–HCl, pH 8.0, and protease inhibitors) and centrifuged at 10,000 rpm 4 °C. The supernatant was collected and stored at -70 °C until use.

Nuclear and non-nuclear cell fractions were obtained following the Abcam protocol. Briefly, the tissue was homogenized with cytoplasmic buffer (250 mM sucrose, 20 mM HEPES pH 7.4, 10 mM KCl, 1.5 mM MgCl_2_, 1 mM EDTA, 1 mM EGTA, 1 mM DTT). The homogenate was centrifuged at 3000 rpm for 5 min, and the supernatant (non-nuclear fraction) was removed. The nuclear pellet was dispersed with a pipette, and then the first step was repeated to remove cytosolic contaminants from the pellet. The nuclear pellet was resuspended in the nuclear buffer (cytoplasmic buffer with 10 % glycerol and 0.1 % SDS). Finally, the nuclear pellet was sonicated on ice and centrifuged at 10,000 rpm, and the supernatant was removed and stored at -70 °C. The protein concentrations were determined using Bradford protein assay. A 6× Laemmli buffer was added to 30 μg of protein, and the sample was loaded in 12 % SDS-polyacrylamide gel and transferred to a PVDF membrane.

The membranes were blocked for 1 h with 5 % of not-fat milk. The membranes were then incubated overnight with primary antibodies to AT_1_R (1:600, rabbit polyclonal AT1 306 antibody, Sc-579), ACE (1:1000; goat polyclonal ACE N-20 antibody, Sc-12184), HO-1 (1:500, rabbit polyclonal HO-1 H-105 antibody, Sc-10789) and γ-GCSc (1:600, rabbit polyclonal γ-GCSc H-338 antibody, Sc-22755) all from Santa Cruz Biotechnology (Delaware Ave, Santa Cruz, CA, USA). HRP-conjugated secondary antibodies (Bio-Rad laboratories, Hercules, CA, USA) were incubated 1 h at a dilution of 1:10,000. Immunoreactivity was detected using ECL western blotting detection reagents (GE health care, Buckinghamshire HP7 9NA, UK). The bands were visualized by exposure to x-ray film. The x-ray film was photodocumented with a UVP (UVP EC3 imaging system, UVP Inc., USA). We used α-actin (donated by Dr. Hernández- Hernández, CINVESTAV-IPN) as an internal control to correct for protein loading. For the subcellular fractions, we used GAPDH (1:1000, mouse monoclonal GAPDH 6C5 antibody, Sc-32,233) and acetyl-Histone-4 (1:1000, mouse monoclonal Ac-Histone H4 Ser 1/Lys 5/Lys 8/Lys 12 antibody, Sc-34,263) to confirm the purity of the fractions.

### [^3^H]-Angiotensin-II binding to lung cell membranes

The lung tissue samples were placed in 10 ml of lysis buffer (10 mM Tris–HCl, 1 mM EGTA, pH 7.4 at 4 °C) and homogenized with a Polytron (3 cycles, 5 s each). The homogenate was centrifuged (1,000×*g*, 10 min, 4 °C), the pellet was discarded and the supernatant was centrifuged at 42,000×*g* (20 min, 4 °C). The resulting pellet was resuspended in lysis buffer and centrifuged again (42,000 × *g*, 20 min, 4 °C). The pellet (membranes) was resuspended in incubation buffer (20 mM Tris–HCl, 5 mM MgCl_2_, 100 mM NaCl, 2 mg/ml bovine serum albumin (BSA); pH 7.4 at 4 °C), and the suspension was immediately used in binding assays. The protein contents were determined by the Bradford assay, using BSA as standard.

Saturation analysis was carried out in 100 μl of incubation buffer containing [Tyrosil-3,5-^3^H (N)]-Angiotensin-II (ARC inc., Saint Louis, MO, USA) (0.5-40 nM) and ~40 μg protein. For single-point experiments, the incubations contained 10 nM [^3^H]-Angiotensin-II. The samples were equilibrated for 60 min at 25 °C in a water bath with continuous gentle rocking, and the incubations were terminated by filtration through Whatman GF/B glass fiber paper, pre-soaked in 0.3 % polyethylenimine. Non-specific binding was determined as the binding insensitive to 100 μM of the antagonist telmisartan (Sigma Aldrich, USA). The filters were washed 3 times with 1 ml ice-cold Tris–HCl buffer (20 mM, pH 7.4), soaked in 3 ml scintillator, and the tritium content was determined by liquid scintillation spectroscopy (Multi-purpose Scintillation Counter, Beckman LS-6500). The saturation binding data were fit with a hyperbolic function using non-linear regression with GraphPad Prism 5 (Graph Pad Software, San Diego, CA, USA).

### Histology

Slides of 5 μm sections of the heart tissues were stained with Hematoxylin/Eosin stain. We photographed all of the intramyocardial coronary arteries observed in the slides (two slides from each tissue sample). We performed a blinded analysis of the measure of the thickness using Image J. Twenty radial measurements for each artery from the intima to the adventitia wall were registered. For the statistical analysis we used the median of each artery; we included in the analysis between 15 to 25 arteries per group. Semi-quantitative analysis of mononuclear cells in heart tissue was performed counting the number of fields with mononuclear cells per slide.

### Statistical analysis

All statistical analyses were performed using SigmaPlot version 11.0. We performed descriptive statistical analyses. To compare the four groups, we performed Kruskal-Wallis test, considering non-normal data distribution. Student Newman-Keul’s post-hoc test was used for all pair-wise comparisons. We considered the differences to be statistically significant when *P* ≤ 0.05. All comparisons were performed with respect to filtered air as the control group.
